# COVID-19–Related Knowledge and Practices Among Health Care Workers in Saudi Arabia: Cross-sectional Questionnaire Study

**DOI:** 10.2196/21220

**Published:** 2021-01-25

**Authors:** Talal Ahmad Shaikhain, Faisal Abdulmohsen Al-Husayni, Essam Awadh Alhejaili, Maha Nawaf Al-Harbi, Anas Abdullah Bogari, Bayan Abdulaziz Baghlaf, Mohammed Saeed Alzahrani

**Affiliations:** 1 Department of Internal Medicine National Guard Hospital Jeddah Saudi Arabia; 2 King Abdullah International Medical Research Center King Abdulaziz Medical City Jeddah Saudi Arabia; 3 Department of Nursing National Guard Hospital Jeddah Saudi Arabia; 4 Department of Infectious Disease National Guard Hospital Jeddah Saudi Arabia; 5 King Saud bin Abdulaziz University for Health Sciences Jeddah Saudi Arabia

**Keywords:** COVID-19, health care workers, infection control, attitude, knowledge, Saudi Arabia

## Abstract

**Background:**

Health care workers are at the front line against COVID-19. The risk of transmission decreases with adequate knowledge of infection prevention methods. However, health care workers reportedly lack a proper attitude and knowledge of different viral outbreaks.

**Objective:**

This study aimed to assess the knowledge and attitude of health care workers in Saudi Arabia toward COVID-19. Assessment of these parameters may help researchers focus on areas that require improvement.

**Methods:**

A cross-sectional questionnaire study was conducted among 563 participants recruited from multiple cities in Saudi Arabia. An online questionnaire was shared via social media applications, which contained questions to health care workers about general information regarding COVID-19 and standard practices.

**Results:**

The mean age of the study population was 30.7 (SD 8) years. Approximately 8.3% (47/563) of the health care workers were isolated as suspected cases of COVID-19, and 0.9% (n=5) were found positive. The majority agreed that social distancing, face masks, and hand washing are effective methods for preventing disease transmission. However, only 63.7% (n=359) knew the correct duration of hand washing. Almost 70% (n=394) strictly adhered to hand hygiene practices, but less than half complied with the practice of wearing a face mask. Significant differences in health care workers' attitudes were observed on the basis of their city of residence, their adherence to COVID-19 practices, and their compliance with the use of a face mask. Among the health care workers, 27.2% (n=153) declared that they will isolate themselves at home and take influenza medication if they experience COVID-19 symptoms.

**Conclusions:**

The majority of health care workers in Saudi Arabia presented acceptable levels of general knowledge on COVID-19, but they lack awareness in some crucial details that may prevent disease spread. Intense courses and competency assessments are highly recommended. Prevention of disease progression is the only option for the time being.

## Introduction

SARS-CoV-2 is a novel virus of the large group of coronaviruses circulating in the environment and is thought to originate from bats [1]. Previous outbreaks such as severe acute respiratory syndrome (SARS) in 2003 and Middle East respiratory syndrome (MERS) in 2015 share similarities with COVID-19 [2]. This novel viral outbreak was epidemiologically linked to the Hua Nan seafood and wet animal wholesale market [3]. Moreover, SARS-CoV-2 was first discovered in Wuhan City, Hubei Province, China, by Chinese authorities. It was initially reported to manifest as pneumonia cases of unknown etiology on December 31, 2019 [4]. Later on, China officially announced the identification of a novel virus, which caused the pneumonia. Shortly after, the World Health Organization (WHO) had declared the outbreak of a novel coronavirus [5]. In February 2020, the disease was named COVID-19 [6].

People infected with COVID-19 may experience a wide range of symptoms, from mild to severe illness. These symptoms include cough, shortness of breath, fever, muscle pain, chills, sore throat, and loss of the sense of taste or smell [7]. However, these symptoms are not universal, as other studies have reported patients with gastrointestinal symptoms such as nausea, vomiting, or diarrhea [7].

According to the WHO, approximately 80% of COVID-19 patients in China experienced mild symptoms and recovered without any medical intervention [8], while 14% of them had experienced severe illness, and 5% were critically ill. However, the risk of having severe illness is higher in the elderly and individuals with underlying chronic diseases such as cancer, diabetes, and lung diseases [8].

Regarding the current state of COVID-19 in Saudi Arabia, the government imposed a curfew from March 23 to June 20, 2020. Mosques, schools, and businesses were closed during that period, and travel was restricted. At the time of writing, Saudi Arabia has reported approximately 49,176 COVID-19 cases, which is lesser than those reported in western countries [9]. In health care settings, all COVID-19 patients were initially hospitalized regardless of disease severity and treated free of charge, including visa violators [10]. Similar to the rest of the world, Saudi Arabia had experienced a shortage of personal protective equipment (PPE), prompting recommendations from the Saudi Center for Disease Prevention and Control on the use and reuse of available PPE [11]. Furthermore, outpatient clinics started seeing most patients virtually, and nonurgent consultations were rescheduled.

According to the Saudi Ministry of Health (SMOH)’s statistical yearbook of 2018, the health care workforce includes 36,717 physicians, 83,616 nurses, 3277 pharmacists, and over 50,000 allied health personnel [12]. Furthermore, health care workers are at the front line and directly come in contact with COVID-19 patients. Consequently, they are always at high risk of infection. The transmission of any disease among health care workers is mainly associated with overcrowding, the absence of isolation facilities, and environmental contamination [13]. However, the transmission risk might also be related to inadequate knowledge of methods for infection prevention [14]. Consequently, health care workers need to have adequate awareness of proper infection prevention practices. In a study conducted at District 2 Hospital, Ho Chi Minh City, Vietnam, the majority (88.4%) of health care workers had adequate knowledge of COVID-19, and 90% of participants have a positive attitude toward COVID-19 [2].

It is essential to have infection control guidelines with the best available evidence to deal with COVID-19 in every health care setting and maximally avoid exposure to the virus. Emphasis should be placed on hand hygiene, which is known to be the best way to prevent the spread of microorganisms and microbial infections in health care facilities [15]. Education on proper PPE, patient screening, and mask use should be provided in accordance with the guidelines of the WHO and the Centers for Disease Control and Prevention (CDC) [16-18]. Previous studies have reported that health care workers might lack a proper attitude and knowledge toward SARS and MERS [19-21]. Therefore, this study aimed to assess the knowledge and attitude toward COVID-19 among health care workers in Saudi Arabia. This assessment may help prevent disease transmission by identifying areas requiring intervention.

## Methods

### Study Design

A cross-sectional questionnaire-based study was performed with health care workers in Saudi Arabia to assess their level of awareness, knowledge, and perception of COVID-19, their level of adherence to the applied curfew, and their understanding of methods for infection prevention. Convenience sampling was carried out by sending the questionnaire through social media platforms (Twitter and WhatsApp), as face-to-face interviews were unavailable owing to curfew regulations. Considering this data collection method, the number of health care workers who received the questionnaire could not be identified because they were encouraged to share the questionnaire within their social circle of health care workers; however, the initial number of health care workers among whom the questionnaire was shared was 1068. The study included health care workers within Saudi Arabia, while those who did not complete the questionnaire or those who worked abroad were excluded. A self-administered questionnaire was developed and distributed from April 30 to May 14, 2020. The questionnaire covered the following items: sociodemographic data such as age, nationality, city of residence, and employment status during the curfew.

Cities were divided as large (population >300,000), medium (population ranging 100,000-300,000), and small (population <100,000) cities. The categorization of cities sizes was based on the measures of the Saudi General Authority for Statistics [22]. The questionnaire also assessed the level of knowledge using “agree,” “neutral,” and “disagree” statements, which also included questions about the duration of hand washing, COVID-19 symptoms, and the timing for COVID-19 testing. Regarding symptoms, the respondents were provided with a list of established COVID-19 symptoms and asked to choose items related to the disease. The Saudi guidelines recommend COVID-19 testing when individuals experience severe respiratory symptoms or flu-like symptoms, or if they come in contact with positive individuals or those with flu-like symptoms. These options were provided to the participants in addition to “any time.” The complete questionnaire is available as Multimedia Appendix 1. After explaining the study objectives to the participants and assuring their confidentiality, the participants were asked to complete the questionnaire. At the end of the questionnaire survey, an email regarding any inquiries was sent to the participants. Informed consent was obtained before data collection, and no identifiers were requested. None of the responders was compensated, and the data were only accessible to the authors to assure confidentiality. The study received ethical approval from the King Abdullah International Medical Research Center (RJ20/079/J).

### Statistical Analysis

Data were entered and analyzed using SPSS (version 25, IBM Corp). Data are presented as ranges, means, SD, medians, and IQR for quantitative variables and frequencies and percentages for qualitative variables. Between-group comparisons were performed using *χ*^2^ or Fisher exact tests. Results are also expressed as odds ratio (OR) and 95% CI values. *P* values less than .05 were considered statistically significant.

## Results

A total of 563 health care workers completed the questionnaire survey. As indicated in Table 1, the participants’ ages ranged from 21 to 69 years. The majority of participants (n=537, 95.4%) were Saudi nationals. Furthermore, 47 (8.3%) health care workers were isolated as suspected COVID-19 cases, and 5 (0.9%) of them tested positive.

Table 2 summarizes the levels of knowledge among the participants, indicated through “agree,” “neutral,” and “disagree” questions. Most of the cohort (n=542, 96.3%) agreed that COVID-19 is a pandemic, while 71.2% (n=401) thought it is more dangerous than seasonal influenza. The highest percentage of agreement (n=547, 97.2%) was obtained for social distancing being an effective method to prevent COVID-19 transmission, followed by hand washing (n=544, 96.6%) and impending curfew (n=542, 96.3%). Furthermore, 33.6% (n=189) of health care workers agreed that COVID-19 transmission could be prevented by wearing gloves.

**Table 1:** Sociodemographic characteristics of health care workers in Saudi Arabia (N=563).

Criterion		Value
**Age (years)**		
	Range	21-69
	Mean (SD)	30.7 (8)
	Median (IQR)	28 (25-33)
**Sex, n (%)**		
	Male	322 (57.2)
	Female	241 (42.8)
**Nationality, n (%)**		
	Saudi	537 (95.4)
	Non-Saudi	26 (4.6)
**City of residence, n (%)**		
	Large	459 (81.5)
	Medium	78 (13.9)
	Small	26 (4.6)
Participants with chronic diseases, n (%)		80 (14.2)
Living with people older than 65 years, n (%)		179 (31.8)
Diagnosed with COVID-19, n (%)		5 (0.9)
Isolated as a suspected case of COVID-19, n (%)		47 (8.3)
**Working status during curfew, n (%)**		
	Yes, I go to work daily	329 (58.4)
	Yes, I work online	142 (25.2)
	No	92 (16.3)

**Table 2:** Levels of knowledge of COVID-19 among health care workers in Saudi Arabia (N=563).

Item	Agree, n (%)	Neutral, n (%)	Disagree, n (%)
COVID-19 is a pandemic	542 (96.3)	14 (2.5)	7 (1.2)
COVID-19 is more dangerous than seasonal influenza	401 (71.2)	105 (18.7)	57 (10.1)
COVID-19 is only dangerous among the elderly and patients with chronic diseases	142 (25.2)	102 (18.1)	319 (56.7)
Hand washing is effective to prevent transmission of COVID-19	544 (96.6)	17 (3)	2 (0.4)
Social distancing is effective to prevent transmission of COVID-19	547 (97.2)	13 (2.3)	3 (0.5)
Wearing face masks is effective to prevent transmission of COVID-19	438 (77.8)	100 (17.8)	25 (4.4)
Wearing hand gloves is effective to prevent transmission of COVID-19	189 (33.6)	172 (30.6)	202 (35.9)
Impending curfew is effective to prevent transmission of COVID-19	520 (92.4)	33 (5.9)	10 (1.8)

When asked about the recommended duration of hand washing to prevent COVID-19 transmission, only 359 (63.8%) of the health care workers selected 40-60 s, while 180 (31.9%) selected 20-30 s, and 24 (4.3%) selected 10-15 s.

**Figure 1:**
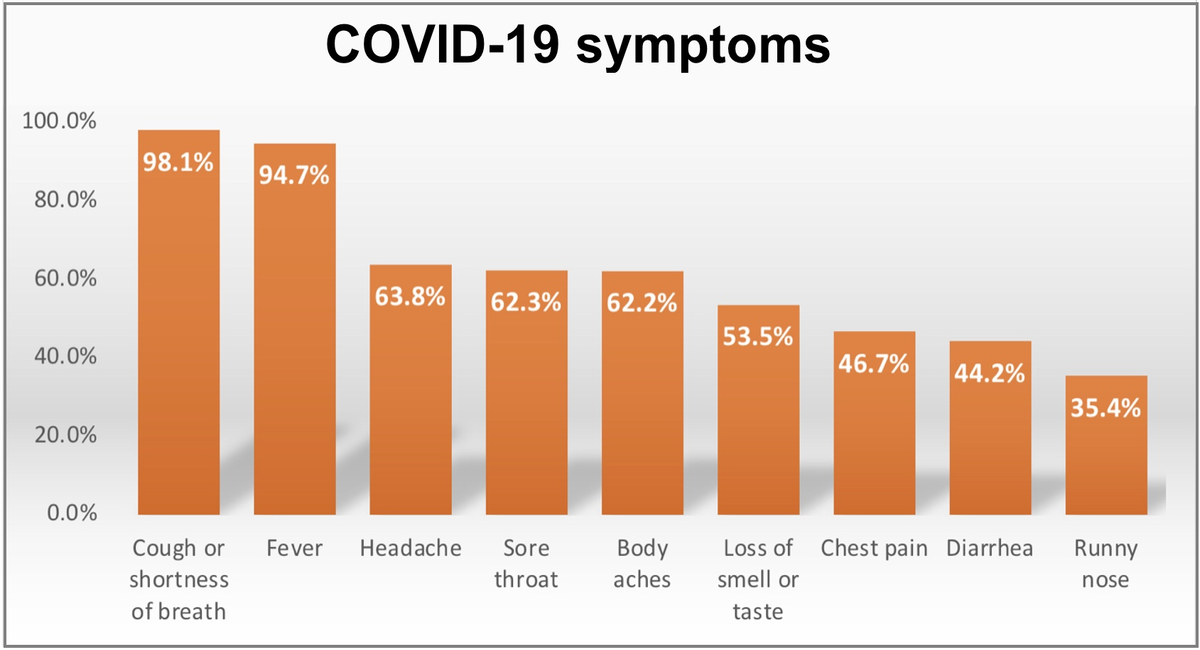
Knowledge of COVID-19 symptoms among health care workers in Saudi Arabia (N=563).

Health care workers were provided a list of symptoms and asked to select those related to COVID-19. As shown in Figure 1, the top selected symptoms were cough or shortness of breath (552/563, 98.1%) and fever (n=533, 94.7%). The lowest percentage (n=199, 35.4%) was for a runny nose.

**Figure 2:**
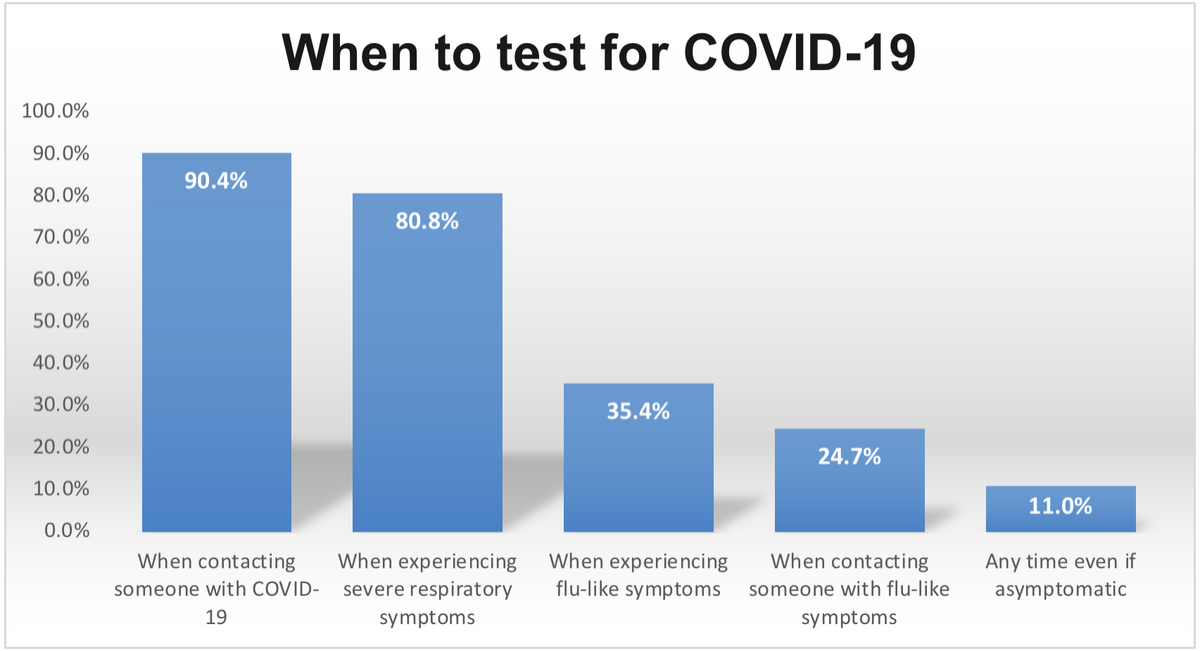
Responses of health care workers in Saudi Arabia regarding the timing for seeking a COVID-19 test (N=563).

Figure 2 shows the responses to the question “when should a person seek testing for COVID-19?” The most frequent response (509/563, 90.4%) was when contacting someone positive for COVID-19, followed by when experiencing severe respiratory symptoms (n=455, 80.8%). Few (n=62, 11%) health care workers chose to test for COVID-19 at any time, even if asymptomatic. Furthermore, 561 (99.6%) health care workers answered “Yes” when asked about the probability of COVID-19 patients being asymptomatic. Moreover, 532 (94.5%) health care workers were aware of the absence of an established therapy for COVID-19.

Table 3 summarizes the attitude of health care workers toward COVID-19. Among them, 461 (81.9%) were always compliant with curfew regulations. Most (n=409, 72.6%) health care workers were always compliant with hand washing. With regards to wearing face masks in public places, 264 (46.9%) health care workers were always compliant, and 116 (20.6%) were not compliant. Furthermore, 290 (51.5%) health care workers frequently followed up with COVID-19–related news.

**Table 3:** Attitudes of health care workers in Saudi Arabia toward COVID-19 (N=563).

Questions		Participants, n (%)
**Are you compliant with curfew regulations?**		
	Always	461 (81.9)
	Most of the time	94 (16.7)
	Sometimes	5 (0.9)
	No	3 (0.5)
**Are you compliant with hand washing?**		
	Always	409 (72.6)
	Most of the time	138 (24.5)
	Sometimes	12 (2.1)
	No	4 (0.7)
**Are you compliant with wearing face masks in public places?**		
	Always	264 (46.9)
	Most of the time	114 (20.2)
	Sometimes	69 (12.3)
	No	116 (20.6)
**Do you follow COVID-19 news?**		
	Always	290 (51.5)
	Most of the time	156 (27.7)
	Sometimes	93 (16.5)
	No	24 (4.3)

When asking the participants what they would do if they experience flu-like symptoms, 350 (62.2%) responded that they would call the SMOH hotline for advice. In comparison, 153 (27.2%) health care workers responded that they would stay at home and take flu medication. Fifty (8.9%) participants responded that they would go to the hospital to test for COVID-19, and 10 (1.8%) would not take any action.

When comparing health care workers living in large, medium, or small cities (Table 4), a significant difference was observed in their compliance with wearing face masks in public places (*P*=.04). The larger the city, the more compliant the participant. Furthermore, health care workers in medium and small cities followed COVID-19 news more than their peers in large cities (*P*=.02).

Finally, 479 (85.1%) health care workers followed COVID-19 news from official authorities including the SMOH and the WHO, while 61 (10.8%) followed news from social media platforms, 19 (3.4%) followed daily news, and only 4 (0.7%) obtained COVID-19 news from their friends.

**Table 4:** Comparison of the attitude toward COVID-19 among health care workers in large, medium, and small cities in Saudi Arabia (N=563).

Questions		City		*P* value	OR^a^ (95% CI)
		Large (N=459), n (%)	Medium/small (N=104), n (%)		
**Are you compliant to curfew regulations?**				.66	0.627 (0.076-5.151)
	Always/most of the time	452 (98.5)	103 (99)		
	Sometimes/no	7 (1.5)	1 (1)		
**Are you compliant to hand washing?**				.49	1.49 (0.471-4.716)
	Always/most of the time	447 (97.4)	100 (96.2)		
	Sometimes/no	12 (2.6)	4 (3.8)		
**Are you compliant to wearing face masks in public places?**				.04^b^	1.574 (1.016-2.438)
	Always/most of the time	317 (69.1)	61 (58.7)		
	Sometimes/no	142 (30.9)	43 (41.3)		
**Do you follow COVID-19 news?**				.02^b^	0.488 (0.262-0.907)
	Always/most of the time	355 (77.3)	91 (87.5)		
	Sometimes/no	104 (22.7)	13 (12.5)		

^a^OR: odds ratio.

^b^Statistically significant (*P*<.05).

## Discussion

### Principal Findings

This study illustrates the knowledge and practices of health care workers in Saudi Arabia at the early stages of the pandemic during a period of significant uncertainty and rapidly changing policies and practices. Among our study participants, marked consensus was observed in their responses to hand hygiene, social distancing, and curfew regulations for effectively preventing disease transmission. Responses to questions on masks and gloves were widely distributed, probably owing to unclear information during the early stages of the pandemic from both the literature and local policies. Moreover, when asked about the timing for COVID-19 testing, most responded with “on experiencing severe symptoms” or “on coming in contact with positive cases,” reflecting the local messaging at that time. Furthermore, their compliance with general hand hygiene and universal masking was concerning and represents an area of improvement. 

When faced with a novel viral pandemic, particularly one with no vaccine or effective treatment at the time of writing, other aspects of disease control become increasingly important. The SMOH implemented daily televised briefings with relevant statistics and discussions regarding the best practices for the current time, and any inquiries usually made by the press were addressed. Practices including hand hygiene and social distancing had the most robust emphasis, while messages regarding the worldwide use of masks were inconsistent owing to their shortage in hospitals and the need to reserve them for frontline health care workers. While the public should be preferentially informed of the best available practices to reduce disease transmission, a higher emphasis should be placed on health care workers, since they constitute a high-risk group for contracting COVID-19, and by the nature of their occupation, they have direct contact with an especially vulnerable part of our community. Hence, it is essential to assess their knowledge and practice and compare them to those of their peers elsewhere. This study also provides an insight into the early stages of the knowledge, attitudes, and practices for disease management among the health care workers, which are expected to change as the pandemic evolves or when more information becomes available.

Multiple outbreaks were reported in health care settings, emphasizing the need for infection control and prevention [23,24]. Risk perception reportedly enhances compliance with protective measures [25]. Approximately 71% of individuals believed that COVID-19 is more dangerous than seasonal influenza, and slightly more than half were aware that COVID-19 could be hazardous to individuals other than the elderly, indicating an area of improvement. Moreover, a study on Egyptian health care workers reported that almost 90% of them believed that the virus is more dangerous in the elderly [26]. Furthermore, Bhagavathula et al [27] reported that only 11.4% of health care workers agreed that COVID-19 is a fatal disease. 

In this study, approximately 8.3% (47/563) of health care workers were isolated as suspected cases of COVID-19; fortunately, only 0.9% (n=5) tested positive, and this number is likely to increase as the spread of the pandemic progresses.

Most of our study participants believe in adopting nationwide protective measures, including social distancing, maintenance of regular hand hygiene, and universal use of face masks during public activities. If these beliefs translate into practice, it could help decrease transmission by decreasing the reproductive number or “flattening the curve,” allowing for better utilization of health care facilities or buying time until vaccine or treatment availability [28,29]. Interestingly, a study form Uganda [30] reported that 55% of health care workers do not believe that face masks may help prevent disease transmission, while almost all of them agreed that avoiding crowded places decreases the risk of acquiring COVID-19. Social distancing proved to be one of the most effective methods of preventing disease transmission during the initial COVID-19 outbreak in Wuhan [31]. 

Regarding hand hygiene, almost all our study participants agreed on the importance of hand washing, which is higher than reported in other studies [27,32], but only 63.8% (n=359) were aware of the correct duration of washing, which is at least 40 s [33].

The primary source of the participants' knowledge was the SMOH daily press briefings and its updates about COVID-19, which contained evidence-based information when available in different areas, including the best infection control practices, policies, and regulations to be implemented and various misconceptions and misinformation about COVID-19. This reflects a drastic improvement in the spread of information alongside practical knowledge through a simple, widely accessible tool such as the television, as opposed to that reported by Khan et al [34] during the MERS outbreak. In their study, the participants faced difficulty following news updates about the disease on the internet from the SMOH website and in looking for new emerging studies. In another study by Albarrak et al [35], the sources of information for the study participants during the MERS outbreak were almost equally distributed among seminars, pamphlets, articles, radio, and television.

We believe that the SMOH performed an admirable job in handling the pandemic and provided transparency and continuous information regarding changes to policies as new data emerged or as the pandemic evolved. Of particular note is the high uniformity in the responses to the messaging, and areas of uncertainty included low levels of knowledge and practices in our study population. We believe that complete transparency and clear messaging are needed for maximum benefits during such events. This study provides a cross-sectional insight into a relatively early stage of the pandemic, and comparisons can potentially be made with the emergence of more data from other countries.

### Limitations

Our study did not define the specialty of the health care workers (eg, nurse, physician, or pharmacist). We also believe that the categorization of health care settings by type (eg, outpatient department, rural hospital, or polyclinic) would have provided more context to the participants' responses.

Furthermore, our study is limited by its convenience sampling method, which might have introduced a potential selection bias. Furthermore, the self-reporting nature of the study questionnaire might have introduced its own set of biases, such as social desirability.

### Conclusion

In conclusion, the majority of our questionnaire respondents had acceptable general knowledge of COVID-19, based on their responses to our questions. Knowledge of decreased disease transmission with the use of face masks was not as uniform as we expected, perhaps reflecting the unclear messaging at that time. Furthermore, approximately half of the study participants disagreed with the statement that COVID-19 is only dangerous in the elderly. Other areas of improvement include the knowledge of the recommended duration of hand washing. Compliance with precautions for infection prevention still need to be emphasized; this can be achieved through intense educational programs and competency assessments to promote positive preventive practices. This study provides a cross-sectional insight into the relatively early stages of the COVID-19 pandemic in Saudi Arabia, and if additional similar studies from other countries become available, comparisons can be made between different populations.

## Appendix

Multimedia Appendix 1 The questionnaire used in the study.

## Abbreviations

CDC: Centers for Disease Control and Prevention
MERS: Middle East respiratory syndrome
OR: odds ratio
SARS: severe acute respiratory syndrome
SMOH: Saudi Ministry of Health
WHO: World Health Organization

## References

[1] Tang X, Wu C, Li X, Song Y, Yao X, Wu X, Duan Y, Zhang H, Wang Y, Qian Z, Cui J (2020). On the origin and continuing evolution of SARS-CoV-2. Natl Sci Rev.

[2] Huynh G, Nguyen TH, Tran V, Vo K, Vo V, Pham L (2020). Knowledge and attitude toward COVID-19 among healthcare workers at District 2 Hospital, Ho Chi Minh City. Asian Pac J Trop Med.

[3] Mackenzie JS, Smith DW (2020). COVID-19: a novel zoonotic disease caused by a coronavirus from China: what we know and what we don't. Microbiol Aust.

[4] (2020). Pneumonia of unknown cause – China. World Health Organization.

[5] (2020). Novel Coronavirus (2019-nCoV): Situation Report 11. World Health Organization.

[6] (2020). Rolling Updates on Coronavirus Disease (COVID-19). World Health Organization.

[7] (2020). Symptoms of Coronavirus. Centers for Disease Control and Prevention.

[8] (2020). Coronavirus disease 2019 (COVID-19): Situation Report 41. World Health Organization.

[9] (COVID-19) Disease Interactive Dashboard. Saudi Center for Disease Prevention and Control.

[10] Coronavirus: Saudi’s King Salman orders treatment for all, including visa violators. Al Arabiya News.

[11] Guidance for Extended Use and Limited Reuse of N95 Respirators. Saudi Center for Disease Prevention and Control.

[12] Statistical Yearbook 2018. Ministry of Health.

[13] Zhang M, Zhou M, Tang F, Wang Y, Nie H, Zhang L, You G (2020). Knowledge, attitude, and practice regarding COVID-19 among healthcare workers in Henan, China. J Hosp Infect.

[14] McEachan R, Taylor N, Harrison R, Lawton R, Gardner P, Conner M (2016). Meta-Analysis of the Reasoned Action Approach (RAA) to Understanding Health Behaviors. Ann Behav Med.

[15] Hand Hygiene Recommendations. Centers for Disease Control and Prevention.

[16] Using Personal Protective Equipment (PPE). Centers for Disease Control and Prevention.

[17] Rational use of personal protective equipment for coronavirus disease (COVID-19) and considerations during severe shortages: interim guidance, 6 April 2020. World Health Organization.

[18] Infection prevention and control during health care when novel coronavirus (nCoV) infection is suspected. World Health Organization.

[19] Deng J, Olowokure B, Kaydos-Daniels S, Chang H, Barwick R, Lee M, Deng C, Factor S, Chiang C, Maloney S, SARS International Field Team (2006). Severe acute respiratory syndrome (SARS): knowledge, attitudes, practices and sources of information among physicians answering a SARS fever hotline service. Public Health.

[20] Althomairy S, Baseer M, Assery M, Alsaffan A (2018). Knowledge and Attitude of Dental Health Professionals about Middle East Respiratory Syndrome in Saudi Arabia. J Int Soc Prev Community Dent.

[21] Alsahafi A, Cheng A (2016). Knowledge, Attitudes and Behaviours of Healthcare Workers in the Kingdom of Saudi Arabia to MERS Coronavirus and Other Emerging Infectious Diseases. Int J Environ Res Public Health.

[22] Population Characteristics surveys 2017. Saudi General Authority for Statistics.

[23] McMichael TM, Currie DW, Clark S, Pogosjans S, Kay M, Schwartz NG, Lewis J, Baer A, Kawakami V, Lukoff MD, Ferro J, Brostrom-Smith C, Rea TD, Sayre MR, Riedo FX, Russell D, Hiatt B, Montgomery P, Rao AK, Chow EJ, Tobolowsky F, Hughes MJ, Bardossy AC, Oakley LP, Jacobs JR, Stone ND, Reddy SC, Jernigan JA, Honein MA, Clark TA, Duchin JS (2020). Epidemiology of Covid-19 in a Long-Term Care Facility in King County, Washington. N Engl J Med.

[24] Pierce M (2020). A short preliminary report on nursing homes and Covid-19: Measures introduced in Ireland. LTC Responses to COVID-19.

[25] Prati G, Pietrantoni L, Zani B (2011). Compliance with recommendations for pandemic influenza H1N1 2009: the role of trust and personal beliefs. Health Educ Res.

[26] Abdel Wahed WY, Hefzy EM, Ahmed MI, Hamed NS (2020). Assessment of Knowledge, Attitudes, and Perception of Health Care Workers Regarding COVID-19, A Cross-Sectional Study from Egypt. J Community Health.

[27] Bhagavathula AS, Aldhaleei WA, Rahmani J, Mahabadi MA, Bandari DK (2020). Knowledge and Perceptions of COVID-19 Among Health Care Workers: Cross-Sectional Study. JMIR Public Health Surveill.

[28] Kissler S, Tedijanto C, Lipsitch M, Grad YH Social distancing strategies for curbing the COVID-19 epidemic. medRxiv..

[29] Dalton CB, Corbett SJ, Katelaris AL (2020). COVID-19: implementing sustainable low cost physical distancing and enhanced hygiene. Med J Aust.

[30] Olum R, Chekwech G, Wekha G, Nassozi DR, Bongomin F (2020). Coronavirus Disease-2019: Knowledge, Attitude, and Practices of Health Care Workers at Makerere University Teaching Hospitals, Uganda. Front Public Health.

[31] Wilder-Smith A, Freedman D (2020). Isolation, quarantine, social distancing and community containment: pivotal role for old-style public health measures in the novel coronavirus (2019-nCoV) outbreak. J Travel Med.

[32] Saqlain M, Munir M, Rehman S, Gulzar A, Naz S, Ahmed Z, Tahir A, Mashhood M (2020). Knowledge, attitude, practice and perceived barriers among healthcare workers regarding COVID-19: a cross-sectional survey from Pakistan. J Hosp Infect.

[33] (2009). World Health Organization.

[34] Khan MU, Shah S, Ahmad A, Fatokun O (2014). Knowledge and attitude of healthcare workers about Middle East Respiratory Syndrome in multispecialty hospitals of Qassim, Saudi Arabia. BMC Public Health.

[35] Albarrak AI, Mohammed R, Al Elayan A, Al Fawaz F, Al Masry M, Al Shammari M, Miaygil SB (2019). Middle East Respiratory Syndrome (MERS): Comparing the knowledge, attitude and practices of different health care workers. J Infect Public Health.

