# Supplementary material for: COVID-19–Related Knowledge and Practices Among Health Care Workers in Saudi Arabia: Cross-sectional Questionnaire Study
Source: JMIR Form Res. 2021 Jan 25;5(1):e21220. doi: 10.2196/21220 (PMC7837509; doi:10.2196/21220)
Supplement: Multimedia Appendix 1 [file formative_v5i1e21220_app1.docx]

Part 1: Socio-demographic Information

1. How old are you?

…………………………..

1. What is your gender?
   - Male
   - Female
2. What is your nationality?
   - Saudi
   - Non-Saudi
3. Where do you live?

………………………….

1. Do you work in the healthcare system?
   - Yes
   - No
2. Do you have any chronic illness?
   - Yes
   - No
3. Do you live with someone who is 65 years old or older?
   - Yes
   - No
4. Have you been diagnosed with COVID-19?
   - Yes
   - No
5. Have you ever been isolated as a suspected case of COVID-19?
   - Yes
   - No
6. Do you still work during the curfew?
   - Yes, I go to work daily
   - Yes, I work online
   - No

Part 2: Knowledge of COVID-19

1. Covid is considered a pandemic.
   - Agree
   - Neutral
   - Disagree
2. COVID-19 is more dangerous than seasonal influenza.
   - Agree
   - Neutral
   - Disagree
3. COVID-19 is only dangerous to elderly and patients with chronic diseases
   - Agree
   - Neutral
   - Disagree
4. Hand washing is effective to prevent transmission of COVID-19
   - Agree
   - Neutral
   - Disagree
5. Social distancing is effective to prevent transmission of COVID-19
   - Agree
   - Neutral
   - Disagree
6. Wearing face masks is effective to prevent transmission of COVID-19
   - Agree
   - Neutral
   - Disagree
7. Wearing hand gloves is effective to prevent transmission of COVID-19
   - Agree
   - Neutral
   - Disagree
8. Impending curfew is effective to prevent transmission of COVID-19
   - Agree
   - Neutral
   - Disagree
9. The correct duration of hand washing to prevent the disease transmission is:
   - 10-15 seconds
   - 20-30 seconds
   - 40-60 seconds
10. Which of the following is a COVID-19 symptom? (You may choose more than one answer):
    - Cough or shortness of breath
    - Fever
    - Body aches
    - Headache
    - Diarrhea
    - Runny nose
    - Sore throat
    - Loss of taste or smell
    - Chest pain
11. When should a person seek testing for COVID-19? (You may choose more than one answer):
    - When contacting with someone who has COVID-19
    - When getting severe respiratory symptoms
    - When getting flu-like symptoms
    - When contacting with someone who has flu-like symptoms
    - Anytime even if asymptomatic
12. Can someone has COVID-19 and be asymptomatic?
    - Yes
    - No
13. Is there an established treatment for COVID-19?
    - Yes
    - No

Part 3: Behaviors toward COVID-19.

1. Are you compliant to curfew regulations?
   - Always
   - Most of the time
   - Sometimes
   - No
2. Are you compliant to hand washing?
   - Always
   - Most of the time
   - Sometimes
   - No
3. Are you compliant to wearing face masks in public places?
   - Always
   - Most of the time
   - Sometimes
   - No
4. Do you follow COVID-19 news?
   - Always
   - Most of the time
   - Sometimes
   - No
5. What is your source of information regarding COVID-19?
   - Official authorities (Saudi Ministry of Health daily briefing or website, World Health Organization, etc..)
   - Daily news
   - Social media (WhatsApp, Twitter, Facebook, etc..)
   - Friends
6. What would you do if you experienced flu-like symptoms?
   - Stay at home and take Flu medications
   - Go to hospital to test for COVID-19
   - Call Saudi Ministry of Health hotline for advice
   - Nothing
